# Associations between chrono-nutrition and chronic kidney disease, urinary incontinence and kidney stones: a cross-sectional study

**DOI:** 10.1016/j.pmedr.2025.103265

**Published:** 2025-10-02

**Authors:** Hao Yu, Zheng Duan, Jiayin Sun, Zhengsen Chen, Zhongqing Wei

**Affiliations:** a Department of Urology, The Second Affiliated Hospital of Nanjing Medical University, Nanjing, China; b Department of Urology, The Second Clinical Medical College of Nanjing Medical University, Nanjing, China

**Keywords:** Chrono-nutrition, Chronic kidney disease, Urinary incontinence, Kidney stones, NHANES, Cross-sectional study

## Abstract

**Objective:**

Dietary timing rhythms may be closely associated with various diseases. This study aims to investigate the relationships between chrono-nutrition and chronic kidney disease (CKD), urinary incontinence, and kidney stones.

**Methods:**

A total of 116,876 participants from 11 cycles of the National Health and Nutrition Examination Survey (1999–2020) were included. Logistic regression, sensitivity analysis, and restricted cubic splines were used to examine the relationships and verify the stability of the results.

**Results:**

We found that a shorter eating window was a risk factor for CKD (OR: 1.10, 95 % CI: 1.03, 1.17), urinary incontinence (OR: 1.11, 95 % CI: 1.03, 1.20), and kidney stones (OR: 1.13, 95 % CI: 1.03, 1.24). Fewer meal frequencies were associated with increased odds of urinary incontinence (twice a day: OR: 1.10, 95 % CI: 1.02, 1.18). Skipping breakfast was associated with a higher likelihood of CKD (OR: 1.10, 95 % CI: 1.02, 1.19) and urinary incontinence (OR: 1.12, 95 % CI: 1.02, 1.23).

**Conclusions:**

Our research indicated that shorter eating window, fewer meal frequencies, and skipping meals may be risk factors for urological diseases. Therefore, the potential risks of fasting regimens used for weight loss or diabetes management should be considered, particularly in individuals with underlying conditions.

## Highlights

• We firstly explored the association between dietary rhythms and urinary health.
• Fasting may improve metabolism but not always be healthy.
• Eating less but more often may be helpful for some kidney diseases.
• Dietary strategies need to be adapted to the individual's constitution.
• It is still recommended to maintain a normal three-meal-a-day eating rhythm.

## 1 Introduction

With abundant global food reserves, the question of when to eat takes on greater significance. Chrono-nutrition, a new paradigm that focuses on the rhythm of eating, is now of widespread concern. It consists of three aspects: timing, frequency, and regularity (Almoosawi et al., 2019). Intermittent fasting, which includes alternate-day fasting and time-restricted eating (TRE), is a key approach. This study focuses mainly on the TRE pattern, where the day's food is consumed within a shorter period, allowing the remainder for fasting (Vasim et al., 2022). Currently, some studies have shown that intermittent fasting may be beneficial for weight loss (Varady et al., 2022), metabolism (Patterson and Sears, 2017), and cardiovascular health (Wirth et al., 2021). Frequency and regularity are equally important for health.

The prevalence of kidney stones among US adults has shown an upward trend over the past several decades, increasing from 3.2 % in 1976–1980 to 8.8 % in 2007–2010 (Stamatelou et al., 2003; Scales Jr. et al., 2012). The worldwide prevalence of chronic kidney disease (CKD) may reach 13.4 % (Hill et al., 2016). According to the National Health and Nutrition Examination Survey (NHANES), 17.1 % of women aged 20 years and above suffer from urinary incontinence (Vaughan and Markland, 2020), while the global prevalence has been estimated to be 8.7 % (Irwin et al., 2006). Urinary incontinence can be further categorized as stress urinary incontinence (SUI), urge urinary incontinence (UUI), and mixed urinary incontinence based on symptoms. Urinary incontinence significantly impacts patients' quality of life and imposes a substantial burden on healthcare systems (Gacci et al., 2022).

Former studies have focused mainly on the composition and nutrients of the diet, with less attention given to temporal rhythms or frequency. Therefore, to alleviate the healthcare burden and reduce the prevalence of urological diseases, we firstly investigate the relationship between them and hope to provide possible recommendations for the general public's dietary habits.

## 2 Materials and methods

### 2.1 Study population

NHANES is a nationally representative survey dataset with samples collected every two years in the United States. This study used data from 11 cycles of NHANES (1999–2020), and only adults aged 20 and above were included. All participants provided written informed consent, and the NHANES protocol was approved by the National Center for Health Statistics Research Ethics Review Board.

### 2.2 Eating window, meal frequency and meal skipping

Eating window was defined as the time interval between the first food intake after 5:00 am and the last intake of the day. This study categorized eating window into less than 11 h, 11–12 h, and more than 12 hours (Palomar-Cros et al., 2023). Meal frequency was defined as the number of meals consumed in a day (including breakfast, lunch/brunch, dinner/supper, or their equivalents in Spanish). Meal frequency was categorized as one, two, three, and four or more meals per day. For meal skipping, this study explored the associations between skipping breakfast or dinner and urological diseases.

### 2.3 Ascertainment of outcomes

CKD was defined as an estimated glomerular filtration rate (eGFR) of <60 mL/min/1.73 m^2^ or a urinary albumin-to-creatinine ratio (uACR) of ≥30 mg/g (KDIGO, 2021). The severity of urinary incontinence was assessed by the questions “How often do you leak urine?” and “How much urine do you lose each time?”. A severity index was calculated by multiplying the two values (range 1–12); an index value greater than or equal to 3 was defined as urinary incontinence (Sandvik et al., 2000). Furthermore, UUI was defined as a “Yes” response to “Urinate before reaching the toilet”, and SUI was defined as a “Yes” response to “Leak urine during physical activities”. Kidney stones were identified by a “Yes” response to the question “Have you ever had kidney stones?”

### 2.4 Covariate assessment

The study adjusted for several covariates, including demographics (age, gender, race, family income level, and education level), body mass index (BMI), sedentary time, smoking status, dietary intake variables (calorie intake, sugar intake, liquid intake, calcium intake, protein intake), serum uric acid levels, and diabetes status (Table S1).

### 2.5 Statistical analysis

Due to the complex sampling design of NHANES, sample weights, clustering, and stratification were accounted for in all analyses. We used R's survey package to handle the complex NHANES weights, applying dietary weights from the first 24-h recall. For the 1999–2016 cycles (WTDRD1) and the 2017–2020 cycle (WTDRD1PP, covering 39 months or 1.625 cycles), weights were calculated as: 1999–2016 = [1/(9 + 1.625)] × WTDRD1; 2017–2020 = [1.625/(9 + 1.625)] × WTDRD1PP. Missing covariate data were imputed using Multiple Imputation by Chained Equations. Continuous variables are presented as means ± standard deviation and compared using *t*-tests; categorical variables are presented as percentages (%) and compared using chi-square tests. We used multiple logistic regression to obtain odds ratios (ORs) and 95 % confidence intervals (CIs). To control for potential confounders, we developed a model that incorporated the relevant covariates. Due to differences in physiological status and dietary habits, a sensitivity analysis excluding pregnant women was conducted to assess the stability of the results. The non-linear relationship between eating window and urological diseases was assessed using restricted cubic splines (RCS) based on a likelihood ratio test, with the median eating window as the reference point (OR = 1). All analyses were conducted using R (version 4.3.1), and a two-sided *p* < 0.05 was considered statistically significant.

## 3 Results

### 3.1 Characteristics of the study population

**Fig. 1:**
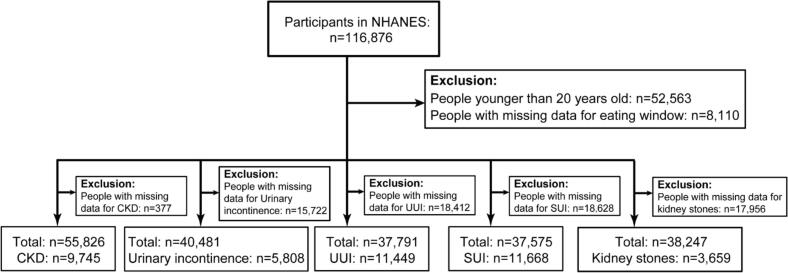
Flowchart of the sample selection for U.S. Adults in NHANES 1999–2020 Abbreviations: NHANES, National Health and Nutrition Examination Survey; CKD, chronic kidney disease; UUI, urge urinary incontinence; SUI, stress urinary incontinence.

The participant selection process is summarized in Fig. 1. After excluding individuals with missing data, the final analytical samples consisted of 55,826 participants for CKD (including 9745 cases), 40,481 for urinary incontinence (5808 cases), 37,575 for SUI (11,668 cases), 37,791 for UUI (11,449 cases), and 38,247 for kidney stones (3659 cases). Baseline characteristics revealed that participants with urological diseases were more likely to be older, non-Hispanic White, have lower income, be overweight, have diabetes, report longer sedentary time, and have lower energy intake (Table 1 & Table S2).

**Table 1:** Baseline characteristics of the U.S. adults (1999–2020) with CKD, urinary incontinence and kidney stones outcome.

Characteristics N (%) / mean ± SD	CKD			Urinary incontinence			Kidney stones		
	Non-CKD	CKD	*p*-value*	Non- Urinary incontinence	Urinary incontinence	*p*-value*	Non-kidney stones	Kidney stones	*p*-value*
N	46,081	9745		34,673	5808		34,588	3659	
Age (years)	47.0 (17.1)	63.0 (16.8)	<0.01	48.0 (17.5)	60.7 (15.5)	0	49.3 (17.7)	56.0 (16.0)	<0.01
Gender:			0.18			<0.01			<0.01
Male	22,302 (48.4 %)	4642 (47.6 %)		18,383 (53.0 %)	1497 (25.8 %)		16,559 (47.9 %)	2022 (55.3 %)	
Female	23,779 (51.6 %)	5103 (52.4 %)		16,290 (47.0 %)	4311 (74.2 %)		18,029 (52.1 %)	1637 (44.7 %)	
Race:			<0.01			<0.01			<0.01
Mexican American	7988 (17.3 %)	1390 (14.3 %)		5213 (15.0 %)	809 (13.9 %)		5030 (14.5 %)	451 (12.3 %)	
Other Hispanic	4040 (8.77 %)	670 (6.88 %)		3377 (9.74 %)	494 (8.51 %)		3528 (10.2 %)	423 (11.6 %)	
Non-Hispanic White	20,019 (43.4 %)	4396 (45.1 %)		14,171 (40.9 %)	2905 (50.0 %)		13,532 (39.1 %)	1959 (53.5 %)	
Non-Hispanic Black	9427 (20.5 %)	2575 (26.4 %)		7815 (22.5 %)	1219 (21.0 %)		8116 (23.5 %)	502 (13.7 %)	
Other Race	4607 (10.00 %)	714 (7.33 %)		4097 (11.8 %)	381 (6.56 %)		4382 (12.7 %)	324 (8.85 %)	
Education level:			<0.01			<0.01			0.40
Less than high school	11,004 (23.9 %)	3227 (33.1 %)		7634 (22.0 %)	1613 (27.8 %)		7891 (22.8 %)	859 (23.5 %)	
High school	10,692 (23.2 %)	2373 (24.4 %)		8034 (23.2 %)	1435 (24.7 %)		8043 (23.3 %)	818 (22.4 %)	
More than high school	24,385 (52.9 %)	4145 (42.5 %)		19,005 (54.8 %)	2760 (47.5 %)		18,654 (53.9 %)	1982 (54.2 %)	
Income level:			<0.01			<0.01			0.03
Low	9231 (20.0 %)	2130 (21.9 %)		6990 (20.2 %)	1279 (22.0 %)		7413 (21.4 %)	717 (19.6 %)	
Median	24,412 (53.0 %)	5734 (58.8 %)		18,443 (53.2 %)	3340 (57.5 %)		18,465 (53.4 %)	2013 (55.0 %)	
High	12,438 (27.0 %)	1881 (19.3 %)		9240 (26.6 %)	1189 (20.5 %)		8710 (25.2 %)	929 (25.4 %)	
BMI (kg/m^2^):			<0.01			<0.01			<0.01
<25	13,701 (29.7 %)	2363 (24.2 %)		10,114 (29.2 %)	1069 (18.4 %)		9871 (28.5 %)	702 (19.2 %)	
25–30	15,663 (34.0 %)	3061 (31.4 %)		11,586 (33.4 %)	1698 (29.2 %)		11,238 (32.5 %)	1205 (32.9 %)	
>30	16,717 (36.3 %)	4321 (44.3 %)		12,973 (37.4 %)	3041 (52.4 %)		13,479 (39.0 %)	1752 (47.9 %)	
Smoking status:			<0.01			<0.01			<0.01
Never smoke	25,669 (55.7 %)	4869 (50.0 %)		19,438 (56.1 %)	2998 (51.6 %)		19,643 (56.8 %)	1824 (49.8 %)	
Former smoker	10,728 (23.3 %)	3201 (32.8 %)		8237 (23.8 %)	1714 (29.5 %)		8085 (23.4 %)	1128 (30.8 %)	
Smoker	9684 (21.0 %)	1675 (17.2 %)		6998 (20.2 %)	1096 (18.9 %)		6860 (19.8 %)	707 (19.3 %)	
Diabetes:			<0.01			<0.01			<0.01
No	42,117 (91.4 %)	6709 (68.8 %)		30,580 (88.2 %)	4437 (76.4 %)		30,179 (87.3 %)	2812 (76.9 %)	
Yes	3964 (8.60 %)	3036 (31.2 %)		4093 (11.8 %)	1371 (23.6 %)		4409 (12.7 %)	847 (23.1 %)	
Sedentary time (min/d)	344 (203)	358 (206)	<0.01	345 (202)	371 (211)	<0.01	347 (203)	357 (207)	<0.01
Total energy intake (kcal/d)	2165 (1021)	1851 (893)	<0.01	2153 (1023)	1889 (854)	<0.01	2109 (1007)	2072 (977)	0.03
Eating window(h)	9.37 (3.66)	9.06 (3.46)	<0.01	9.40 (3.67)	9.20 (3.44)	<0.01	9.37 (3.65)	9.40 (3.48)	0.62
Meal frequency (times/d):			<0.01			<0.01			<0.01
1	23,989 (52.1 %)	5239 (53.8 %)		18,043 (52.0 %)	3134 (54.0 %)		17,933 (51.8 %)	1960 (53.6 %)	
2	2684 (5.82 %)	529 (5.43 %)		1980 (5.71 %)	277 (4.77 %)		1977 (5.72 %)	167 (4.56 %)	
3	12,544 (27.2 %)	2759 (28.3 %)		9383 (27.1 %)	1586 (27.3 %)		9237 (26.7 %)	995 (27.2 %)	
≥4	6864 (14.9 %)	1218 (12.5 %)		5267 (15.2 %)	811 (14.0 %)		5441 (15.7 %)	537 (14.7 %)	
Skip breakfast:			<0.01			<0.01			<0.01
No	38,643 (83.9 %)	8478 (87.0 %)		29,113 (84.0 %)	5090 (87.6 %)		29,126 (84.2 %)	3163 (86.4 %)	
Yes	7438 (16.1 %)	1267 (13.0 %)		5560 (16.0 %)	718 (12.4 %)		5462 (15.8 %)	496 (13.6 %)	
Skip dinner:			0.11			1			0.39
No	41,555 (90.2 %)	8736 (89.6 %)		31,343 (90.4 %)	5250 (90.4 %)		31,294 (90.5 %)	3294 (90.0 %)	
Yes	4526 (9.82 %)	1009 (10.4 %)		3330 (9.60 %)	558 (9.61 %)		3294 (9.52 %)	365 (9.98 %)	
Sugar intake (gm/d)	117 (80.7)	99.2 (69.0)	<0.01	113 (79.7)	103 (67.1)	<0.01	110 (77.6)	112 (81.4)	0.17
Liquid intake (gm/d)	2709 (1488)	2401 (1405)	<0.01	2934 (1535)	2649 (1357)	<0.01	2881 (1515)	2834 (1496)	0.07
Calcium intake (mg/d)	913 (592)	797 (524)	<0.01	929 (589)	853 (533)	<0.01	917 (581)	902 (577)	0.12
Protein intake (gm/d)	82.3 (43.4)	71.5 (37.9)	<0.01	82.5 (43.7)	70.7 (35.3)	<0.01	80.7 (42.8)	78.1 (41.5)	<0.01
Serum uric acid (μmol/L)	315 (82.2)	357 (101)	<0.01	324 (86.2)	324 (89.7)	0.88	323 (86.2)	335 (90.8)	<0.01

N (%) for categorical variables. Mean ± SD for continuous variables. *p-value were calculated by t-test and chi-square test.

Abbreviations: CKD, chronic kidney disease; SD, standard deviation; BMI, body mass index.

### 3.2 Associations between eating window and urological diseases

**Fig. 2:**
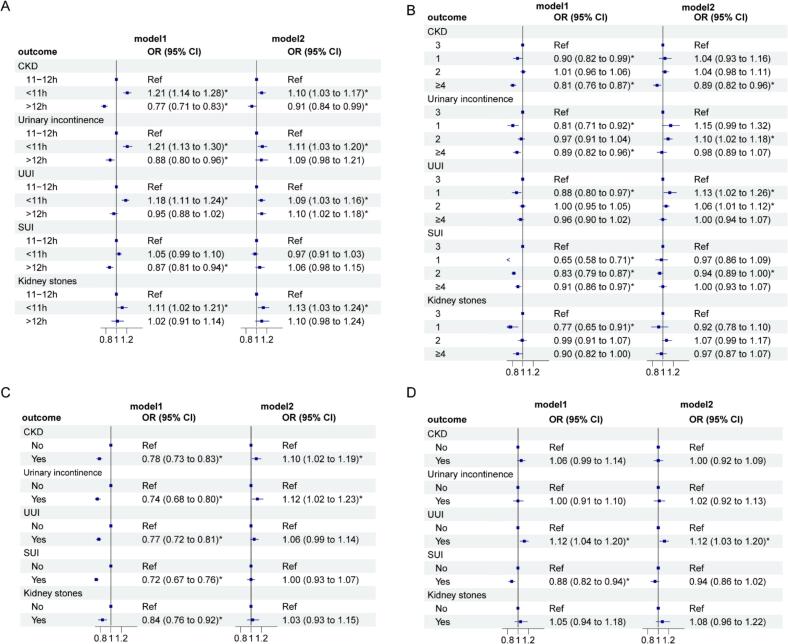
Associations between eating window(a), meal frequency (b), skipping breakfast (c) and skipping dinner (d) and urological diseases among U.S. adults (1999–2020). Model 1 is unadjusted for covariates. Model 2 is adjusted for the following covariates: age, gender, race, family income level, education level, BMI, sedentary time, smoking status, calorie intake, sugar intake, liquid intake, calcium intake, protein intake, serum uric acid, and diabetes status. Abbreviations: CKD, chronic kidney disease; UUI, urge urinary incontinence; SUI, stress urinary incontinence; BMI, body mass index

The correlations between eating window and urological diseases are shown in Fig. 2a. Compared to participants with an eating window of 11–12 h per day, those who ate for less than 11 h per day had higher odds of developing CKD (OR: 1.10, 95 % CI: 1.03, 1.17), urinary incontinence (OR: 1.11, 95 % CI: 1.03, 1.20), and kidney stones (OR: 1.13, 95 % CI: 1.03, 1.24). For the two urinary incontinence subtypes, shorter eating window was a risk factor for UUI (OR: 1.09, 95 % CI: 1.03, 1.16) but not for SUI (OR: 0.97, 95 % CI: 0.91, 1.03). An eating window longer than 12 h was associated with lower odds of CKD (OR: 0.91, 95 % CI: 0.84, 0.99) and higher odds of UUI (OR: 1.10, 95 % CI: 1.02, 1.18).

### 3.3 Associations between meal frequency and urological diseases

We found that individuals who ate one meal a day had increased odds of UUI (OR: 1.13, 95 % CI: 1.02, 1.26) compared to those who ate three times a day, but no significant association was observed for CKD, urinary incontinence, SUI, or kidney stones. Eating two meals per day was associated with higher odds of urinary incontinence (OR: 1.10, 95 % CI: 1.02, 1.18) and UUI (OR: 1.06, 95 % CI: 1.01, 1.12), but lower odds of SUI (OR: 0.94, 95 % CI: 0.89, 0.99). Eating four or more meals per day was associated with reduced odds of CKD (OR: 0.89, 95 % CI: 0.82, 0.96). No other significant differences were found for other conditions (Fig. 2b).

### 3.4 Associations between meal skipping and urological diseases

Skipping breakfast was associated with increased odds of developing CKD (OR: 1.10, 95 % CI: 1.02, 1.19) and urinary incontinence (OR: 1.12, 95 % CI: 1.02, 1.23) compared to those who ate breakfast (Fig. 2c). Skipping dinner was associated with increased odds of UUI (OR: 1.12, 95 % CI: 1.03, 1.20). No significant differences were found for other conditions (Fig. 2d).

### 3.5 Sensitivity analysis

After excluding pregnant participants (*n* = 448), the results remained unchanged (Fig. S1–3).

### 3.6 Non-linear association between eating window and the risk of urological diseases

**Fig. 3:**
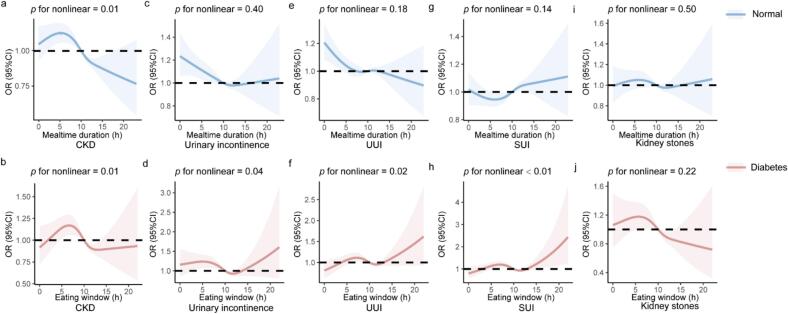
Restricted Cubic Splines for the associations between eating window and urological diseases by diabetes status among U.S. adults (1999–2020). The analysis was adjusted for age, gender, race, family income level, education level, BMI, sedentary time, smoking status, calorie intake, sugar intake, liquid intake, calcium intake, protein intake and serum uric acid. Abbreviations: CKD, chronic kidney disease; UUI, urge urinary incontinence; SUI, stress urinary incontinence; BMI, body mass index

Non-linear associations were observed between eating window and four urological diseases in individuals with diabetes (CKD: *p* for nonlinear = 0.01, urinary incontinence: *p* for nonlinear = 0.04, UUI: *p* for nonlinear = 0.02, SUI: *p* for nonlinear < 0.01). Among participants without diabetes, a significant non-linear association was observed only for CKD (*p* for nonlinear = 0.01). When meals were consumed within 11–12 h, individuals with diabetes had lower odds of these diseases, especially urinary incontinence, compared to those without diabetes (Fig. 3).

## 4 Discussion

Our study indicated a potential association between chrono-nutrition and urological diseases. Regarding eating window, a shorter duration was a risk factor for the three main outcomes. In terms of meal frequency, eating fewer than three meals per day increased the odds of urinary incontinence and kidney stones, while eating more than three meals was a protective factor for CKD. Regarding meal skipping, skipping breakfast was associated with increased odds of CKD and urinary incontinence, while skipping dinner was associated with increased odds of UUI.

There are similarities between our results and previous studies. Our finding that fewer meals was a risk factor for urinary incontinence and kidney stones, while more than three meals reduced CKD odds, suggests that frequent, small meals may be beneficial. Schoenfeld et al. found an inverse relationship between eating frequency and adiposity (Schoenfeld et al., 2015). However, St-Onge et al. noted conflicting conclusions about meal frequency. Additionally, our result that skipping breakfast increases CKD and urinary incontinence odds is consistent with Rong et al. and St-Onge et al., who found skipping breakfast associated with higher cardiovascular mortality (St-Onge et al., 2017; Rong et al., 2019).

Notably, our finding that a shorter eating window increases the odds of urological diseases challenges the prevailing view that intermittent fasting (e.g., 16:8 pattern) is beneficial. For example, Yang et al. reported intermittent fasting may mitigate diabetic nephropathy (Yang et al., 2022), contrary to our conclusion. Few studies share our perspective; Cienfuegos et al. found prolonged fasting can cause harmful hormonal fluctuations (Cienfuegos et al., 2022), and Costa et al. advised against fasting for CKD patients due to risks of dehydration and disease progression (Costa et al., 2021). Therefore, although many studies show benefits of shortened eating window, more attention is needed to its potential risks.

As a new field in nutritional sciences, the mechanism of chrono-nutrition is closely related to the biological clock and circadian rhythms. The human biological clock is comprised of two distinct components: the core clock, which is distributed in the supraoptic nucleus, and the peripheral clock, which is distributed in other peripheral organs and tissues (Ahluwalia, 2022). In consideration of time rhythms, fasting is an inevitable point of discussion. Fasting has been shown to improve insulin sensitivity, blood pressure, and oxidative stress. It can also enhance mitochondrial health, gene repair and autophagy, as well as promote stem cell-based regeneration (Sutton et al., 2018; He et al., 2022; Mattson et al., 2017). Nevertheless, irrespective of the dietary pattern, modifying the established eating frequency, influencing the circadian rhythm and sleep, may result in endocrine disruption and an imbalance of the biological clock. This, in turn, gives rise to a cascade of metabolic and immune-validating reactions, culminating in a state of hormonal imbalance within the body. Such occurrences may result in the development of disease (Reinke and Asher, 2019).

CKD patients have a lower metabolic function for nutrients due to the destruction of their renal function, which produces a systemic inflammatory response and hormone deficiencies (Ikizler et al., 2013). The effects of fasting, meal skipping, and disruption of circadian clock balance, which result in widespread activation of the systemic immune system, exacerbate this response and further increase the odds of disease in the general population (Costa et al., 2021). Although Yang's study demonstrated that intermittent fasting can reduce renal tubulointerstitial fibrosis by maintaining mitochondrial homeostasis through the regulation of several aspects of mitochondrial density and respiratory capacity (Yang et al., 2022), it is unclear whether this response would exacerbate oxidative damage and thus promote CKD formation, as different tissues respond differently.

For kidney stones, known risk factors include liquid intake (Siener, 2021), dietary minerals, and serum uric acid, which we adjusted for in our model. The persistent association with shorter eating window suggests an independent role for temporal eating patterns. Consequently, fasting may reduce the kidneys' metabolic activity, impairing the processing of fluids and minerals like calcium and phosphorus. This can lead to increased concentrations of these substances in the urine. Their subsequent accumulation at the renal papillae may then promote the formation of stones (Zhou et al., 2024; Khan et al., 2016).

Indeed, it is difficult to generalize across diseases due to their different pathological mechanisms, which is why we divided urinary incontinence into SUI and UUI. SUI is more common in middle-aged and elderly women due to their physiological structure and pelvic floor dysfunction caused by estrogen withdrawal during perimenopause (Wu, 2021). Many studies have shown that metabolic syndrome (Fwu et al., 2024), diabetes, and obesity accelerate the occurrence and development of SUI. Appropriate meal reduction and fasting can control body weight and improve metabolism (Varady et al., 2022; Patterson and Sears, 2017; He et al., 2022), which to some extent protects against SUI, consistent with our findings.

In contrast, UUI prevalence does not differ significantly by gender. There are numerous potential causes for UUI, including alterations in bladder coordination and the functional anatomy of the urinary tract (Nandy and Ranganathan, 2022). Neurological factors also play a key role (Panicker, 2020). Our findings indicate that reduced meal frequency and skipping breakfast or dinner may increase the odds of UUI. This suggests that extended fasting may be detrimental, potentially due to disruptions in nutritional and metabolic balance and the activation of inflammatory responses (Marko et al., 2024). These factors may negatively impact hormonal and neuromodulator responses, influencing UUI progression. This correlation was more pronounced in older men with diabetes, which may be attributed to more significant diabetic peripheral neuropathy, leading to bladder detrusor fibrosis and urothelial dysfunction (Wang et al., 2020).

The strengths of our study include the use of NHANES data, which provides a representative sample of US adults, enhancing the generalizability of the conclusions. Additionally, since the physiological mechanisms of these diseases are markedly different, we analyzed them separately based on three aspects of chrono-nutrition. Furthermore, we adjusted for a variety of potential confounding variables and constructed the model for validation; sensitivity analyses, and RCS were also used to verify the stability of the results. Finally, to our knowledge, this study is the first to investigate the relationship between dietary timing and urological disease, providing some new insights for clinical dietitians and urologists.

However, the study's observational design prevents the establishment of causality. Secondly, the data in this study was mostly self-reported by participants, which may introduce recall and reporting bias. Additionally, with the diverse and individualized diets in modern society, it is difficult for our measures to fully represent the complex eating behaviors of free-living individuals.

## 5 Conclusions

This study indicated that shorter eating window (< 11 h), fewer meal frequencies (< 3), and skipping meals may be risk factors for CKD, urinary incontinence, and kidney stones. These findings partially agree with but also challenge the previously advocated view of the intermittent fasting pattern, necessitating more research on its effects on other body systems. Although short-term benefits of fasting for metabolic and cardiovascular health exist, it is recommended that individuals, especially those with underlying conditions, maintain a normal three-meal-a-day pattern, avoid skipping meals or prolonged fasting, and ensure proper nutrition. This study also highlights the need for the public to choose appropriate dietary patterns.

## CRediT authorship contribution statement

**Hao Yu:** Writing – original draft, Software, Formal analysis, Conceptualization. **Zheng Duan:** Writing – review & editing, Validation, Data curation. **Jiayin Sun:** Writing – original draft, Methodology, Investigation. **Zhengsen Chen:** Writing – review & editing, Supervision, Conceptualization. **Zhongqing Wei:** Writing – review & editing, Supervision, Funding acquisition.

## Consent for publication

Not applicable.

## Ethics approval and consent to participate

NHANES was consistent with approval from the National Center for Health Statistics Research Ethics Review Board.

## Funding

This work was supported by the by 10.13039/501100001809National Natural Science Foundation of China (NSFC: 82370781).

## Declaration of competing interest

The authors declare that they have no known competing financial interests or personal relationships that could have appeared to influence the work reported in this paper.

## Data Availability

Data will be made available on request.

## Appendix A Supplementary data

Supplementary tables and figures. Image 1
