# Supplementary material for: Associations between chrono-nutrition and chronic kidney disease, urinary incontinence and kidney stones: a cross-sectional study
Source: Prev Med Rep. 2025 Oct 2;59:103265. doi: 10.1016/j.pmedr.2025.103265 (PMC12538022; doi:10.1016/j.pmedr.2025.103265)
Supplement: Supplementary file 1 — Supplementary tables and figures. [file mmc1.docx]

**Table S1: Types and assessment principles of covariates.**

| Variables | Variable types | The corresponding content in NHANES and classification principles |
| --- | --- | --- |
| Age | Continuous variables | Age at screening adjudicated/Age in years at screening |
| Sedentary time | Continuous variables | Minutes sedentary activity |
| Calorie intake | Continuous variables | Dietary Interview - Individual Foods, First Day, Energy (kcal) |
| Sugar intake | Continuous variables | Dietary Interview - Individual Foods, First Day, Total sugars (gm) |
| Liquid intake | Continuous variables | Dietary Interview - Individual Foods, First Day, Moisture (gm) |
| Calcium intake | Continuous variables | Dietary Interview - Individual Foods, First Day, Calcium (mg) |
| Protein intake | Continuous variables | Dietary Interview - Individual Foods, First Day, Protein (gm) |
| Serum uric acid | Continuous variables | Standard Biochemistry Profile, Uric acid (umol/L) |
| Gender | Categorical variables | Gender: Male vs. Female |
| Race | Categorical variables | Race/Ethnicity - Recode: Mexican American, Other Hispanic, Non-Hispanic White, Non-Hispanic Black, and Other Race |
| Family income level | Categorical variables | According to the definition of ratio of family income to poverty, low: < 1, medium: 1–4, high: > 4) |
| BMI | Categorical variables | Body Mass Index: Normal: < 25, overweight: 25**–**30, obesity: > 30 |
| Smoking status | Categorical variables | Participants were categorized by smoking status based on two criteria: lifetime consumption of at least 100 cigarettes and current smoking behavior. The categories included:   1. Never smokers (those who had smoked fewer than 100 cigarettes in their lifetime and were not currently smoking)   2. Former smokers (those who had smoked at least 100 cigarettes but were not currently smoking)  3. Current smokers (those who reported current smoking, regardless of lifetime cigarette consumption) |
| Diabetes | Categorical variables | Doctor told you have diabetes: Yes vs. No |

**Table S2: Baseline characteristics of the U.S. adults (1999–2020) with UUI and SUI outcome.**

| Characteristics  N (%) / mean ± SD | UUI | | |  | SUI | | |
| --- | --- | --- | --- | --- | --- | --- | --- |
|  | Non UUI | UUI | *p*-value* |  | Non SUI | SUI | *p*-value* |
| N | 37,791 | 11,449 |  |  | 37,575 | 11,668 |  |
| Age (years) | 47.2 (17.6) | 59.0 (16.4) | 0 |  | 48.5 (18.2) | 54.4 (16.7) | <0.01 |
| Gender: |  |  | <0.01 |  |  |  | 0 |
| Male | 20,137 (53.3 %) | 3962 (34.6 %) |  |  | 22,783 (60.6 %) | 1319 (11.3 %) |  |
| Female | 17,654 (46.7 %) | 7487 (65.4 %) |  |  | 14,792 (39.4 %) | 10,349 (88.7 %) |  |
| Race: |  |  | <0.01 |  |  |  | <0.01 |
| Mexican American | 6083 (16.1 %) | 1714 (15.0 %) |  |  | 5864 (15.6 %) | 1934 (16.6 %) |  |
| Other Hispanic | 3267 (8.64 %) | 919 (8.03 %) |  |  | 3187 (8.48 %) | 1000 (8.57 %) |  |
| Non-Hispanic White | 16,651 (44.1 %) | 5140 (44.9 %) |  |  | 15,965 (42.5 %) | 5830 (50.0 %) |  |
| Non-Hispanic Black | 7704 (20.4 %) | 2968 (25.9 %) |  |  | 8721 (23.2 %) | 1948 (16.7 %) |  |
| Other Race | 4086 (10.8 %) | 708 (6.18 %) |  |  | 3838 (10.2 %) | 956 (8.19 %) |  |
| Education level: |  |  | <0.01 |  |  |  | <0.01 |
| Less than high school | 8641 (22.9 %) | 3163 (27.6 %) |  |  | 8868 (23.6 %) | 2938 (25.2 %) |  |
| High school | 8809 (23.3 %) | 2770 (24.2 %) |  |  | 8905 (23.7 %) | 2675 (22.9 %) |  |
| More than high school | 20,341 (53.8 %) | 5516 (48.2 %) |  |  | 19,802 (52.7 %) | 6055 (51.9 %) |  |
| Income level: |  |  | <0.01 |  |  |  | <0.01 |
| Low | 7384 (19.5 %) | 2435 (21.3 %) |  |  | 7430 (19.8 %) | 2385 (20.4 %) |  |
| Median | 20,097 (53.2 %) | 6499 (56.8 %) |  |  | 20,183 (53.7 %) | 6417 (55.0 %) |  |
| High | 10,310 (27.3 %) | 2515 (22.0 %) |  |  | 9962 (26.5 %) | 2866 (24.6 %) |  |
| BMI (kg/m^2^ ): |  |  | <0.01 |  |  |  | <0.01 |
| <25 | 11,468 (30.3 %) | 2469 (21.6 %) |  |  | 11,283 (30.0 %) | 2655 (22.8 %) |  |
| 25-30 | 13,023 (34.5 %) | 3433 (30.0 %) |  |  | 12,985 (34.6 %) | 3474 (29.8 %) |  |
| >30 | 13,300 (35.2 %) | 5547 (48.4 %) |  |  | 13,307 (35.4 %) | 5539 (47.5 %) |  |
| Smoking status: |  |  | <0.01 |  |  |  | <0.01 |
| Never smoke | 21,061 (55.7 %) | 5817 (50.8 %) |  |  | 20,322 (54.1 %) | 6555 (56.2 %) |  |
| Former smoker | 8918 (23.6 %) | 3410 (29.8 %) |  |  | 9425 (25. 1%) | 2903 (24.9 %) |  |
| Smoker | 7812 (20.7 %) | 2222 (19.4 %) |  |  | 7828 (20.8 %) | 2210 (18.9 %) |  |
| Diabetes: |  |  | <0.01 |  |  |  | <0.01 |
| No | 33,857 (89.6 %) | 9023 (78.8 %) |  |  | 33,098 (88.1 %) | 9783 (83.8 %) |  |
| Yes | 3934 (10.4 %) | 2426 (21.2 %) |  |  | 4477 (11.9 %) | 1885 (16.2 %) |  |
| Sedentary time (min/d) | 346 (203) | 358 (207) | <0.01 |  | 348 (204) | 351 (203) | 0.09 |
| Total energy intake (kcal/d) | 2164 (1029) | 1964 (911) | <0.01 |  | 2188 (1045) | 1893 (827) | <0.01 |
| Mealtime duration (h) | 9.36 (3.67) | 9.23 (3.49) | <0.01 |  | 9.31 (3.72) | 9.40 (3.35) | <0.01 |
| Meal frequency (times/d): |  |  | 0.04 |  |  |  | <0.01 |
| 1 | 19,720 (52.2 %) | 6058 (52.9 %) |  |  | 19,258 (51.3 %) | 6521 (55.9 %) |  |
| 2 | 2200 (5.82 %) | 597 (5.21 %) |  |  | 2294 (6.11 %) | 502 (4.30 %) |  |
| 3 | 10,283 (27.2 %) | 3153 (27.5 %) |  |  | 10,500 (27.9 %) | 2942 (25.2 %) |  |
| ≥4 | 5588 (14.8 %) | 1641 (14.3 %) |  |  | 5523 (14.7 %) | 1703 (14.6 %) |  |
| Skip breakfast: |  |  | <0.01 |  |  |  | <0.01 |
| No | 31,660 (83.8 %) | 9972 (87.1 %) |  |  | 31,408 (83.6 %) | 10,230 (87.7 %) |  |
| Yes | 6131 (16.2 %) | 1477 (12.9 %) |  |  | 6167 (16.4 %) | 1438 (12.3 %) |  |
| Skip dinner: |  |  | <0.01 |  |  |  | <0.01 |
| No | 34,197 (90.5 %) | 10,246 (89.5 %) |  |  | 3,3813 (90.0 %) | 10,632 (91.1 %) |  |
| Yes | 3594 (9.51 %) | 1203 (10.5 %) |  |  | 3762 (10.0 %) | 1036 (8.88 %) |  |
| Sugar intake (gm/d) | 115 (80.6) | 107 (71.7) | <0.01 |  | 116 (81.4) | 105 (68.6) | <0.01 |
| Liquid intake (gm/d) | 2765 (1513) | 2583 (1417) | <0.01 |  | 2769 (1531) | 2575 (1351) | <0.01 |
| Calcium intake (mg/d) | 919 (597) | 850 (532) | <0.01 |  | 921 (599) | 847 (526) | <0.01 |
| Protein intake (gm/d) | 82.7 (44.0) | 74.0 (37.5) | <0.01 |  | 83.7 (44.5) | 70.8 (34.4) | <0.01 |
| Serum uric acid (μmol/L) | 324 (86.5) | 323 (88.8) | 0.67 |  | 330 (87.0) | 304 (84.1) | <0.01 |

N (%) for categorical variables. Mean ± SD for continuous variables. **p*-value were calculated by t-test and chi-square test.

Abbreviations: UUI, urge urinary incontinence; SUI, stress urinary incontinence; SD, standard deviation; BMI, body mass index.


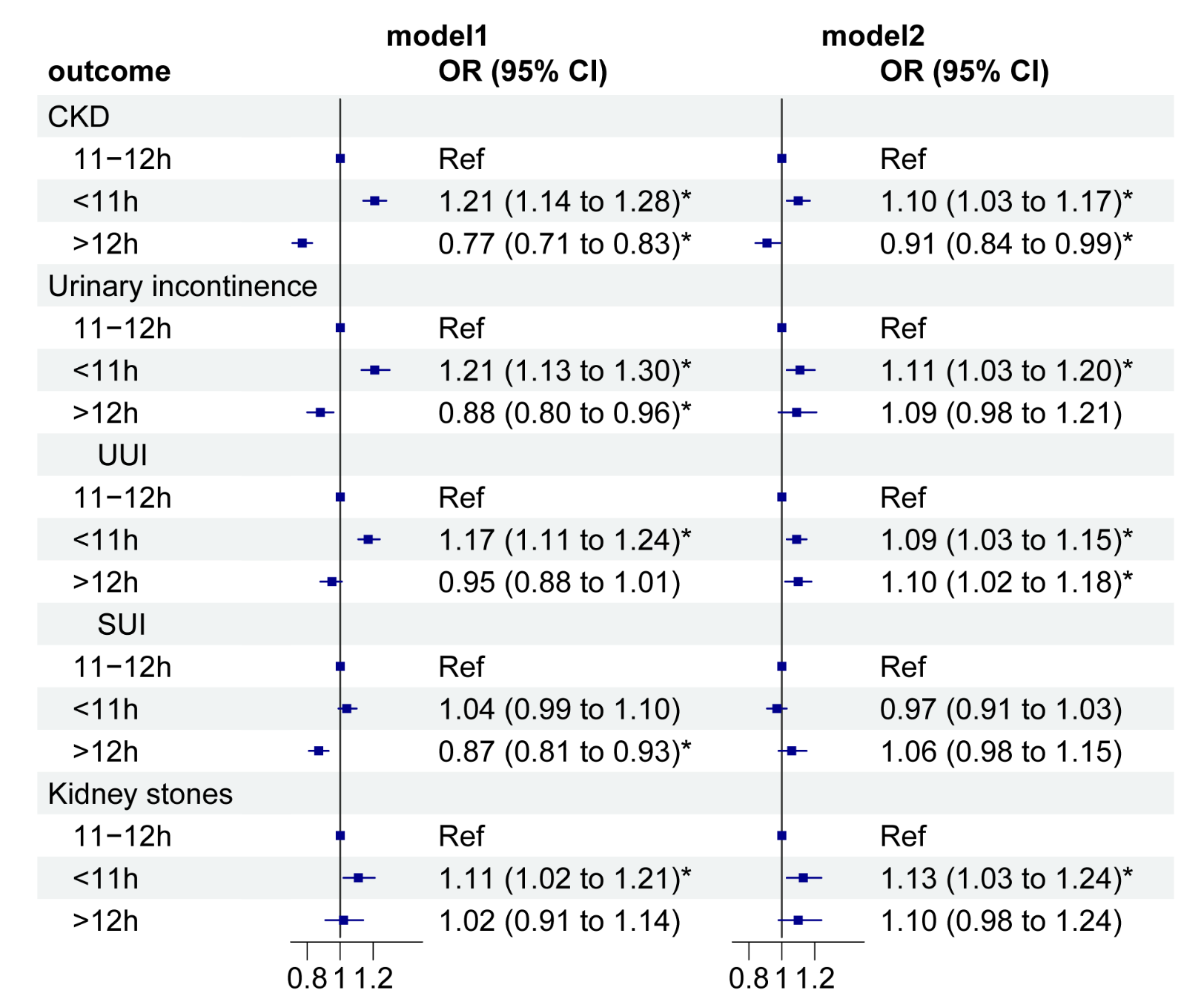
**Fig. S1. Associations between eating window and urological diseases** **among U.S. adults (1999–2020) after excluding pregnant participants.**

Model 1 is unadjusted for covariates

Model 2 is adjusted for the following covariates: age, gender, race, family income level, education level, BMI, sedentary time, smoking status, calorie intake, sugar intake, liquid intake, calcium intake, protein intake, serum uric acid, and diabetes status

Abbreviations: CKD, chronic kidney disease; UUI, urge urinary incontinence; SUI, stress urinary incontinence; BMI, body mass index


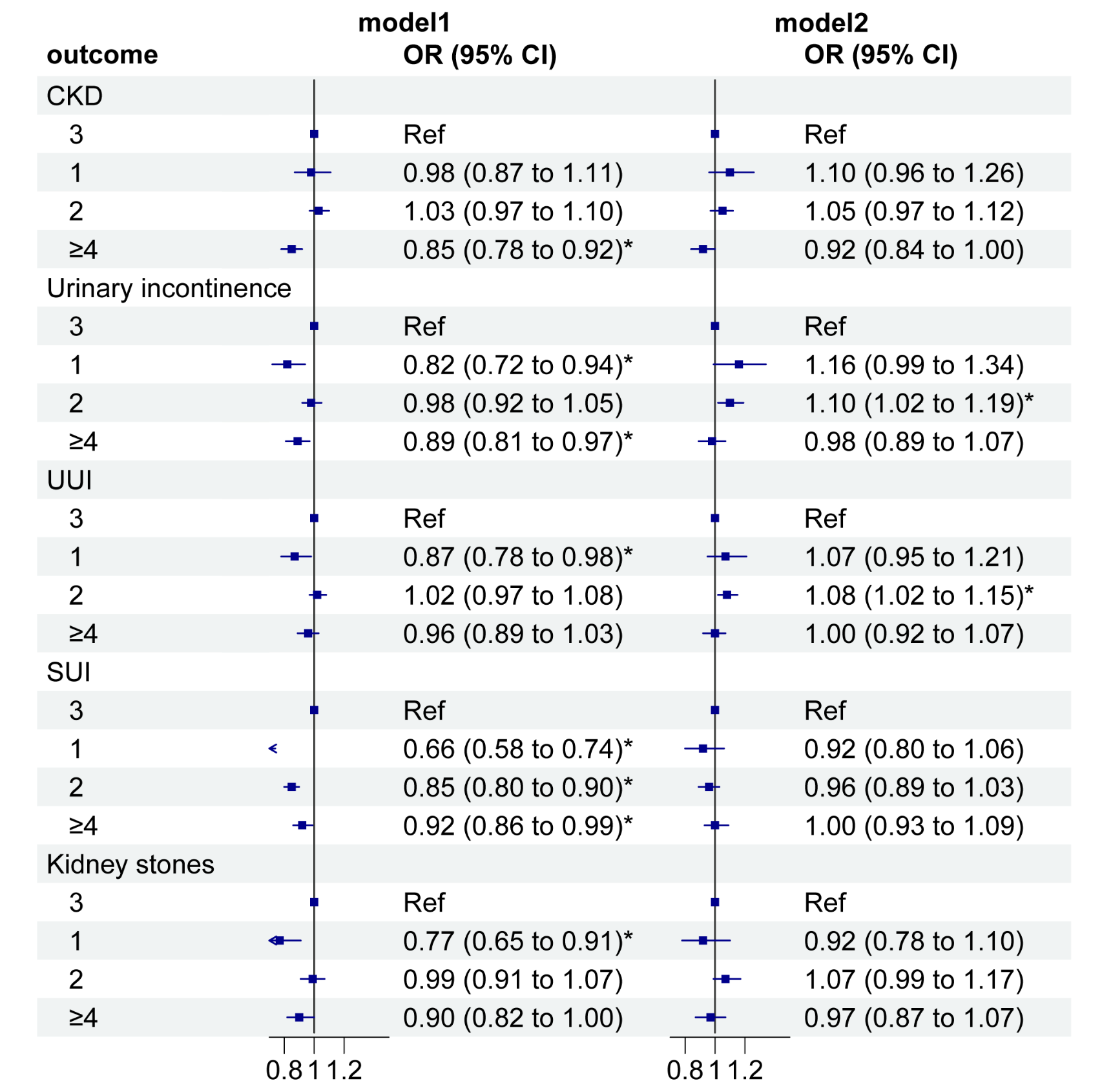
**Fig. S2. Associations between meal frequency and urological diseases among U.S. adults (1999–2020) after excluding pregnant participants.**

Model 1 is unadjusted for covariates

Model 2 is adjusted for the following covariates: age, gender, race, family income level, education level, BMI, sedentary time, smoking status, calorie intake, sugar intake, liquid intake, calcium intake, protein intake, serum uric acid, and diabetes status

Abbreviations: CKD, chronic kidney disease; UUI, urge urinary incontinence; SUI, stress urinary incontinence; BMI, body mass index


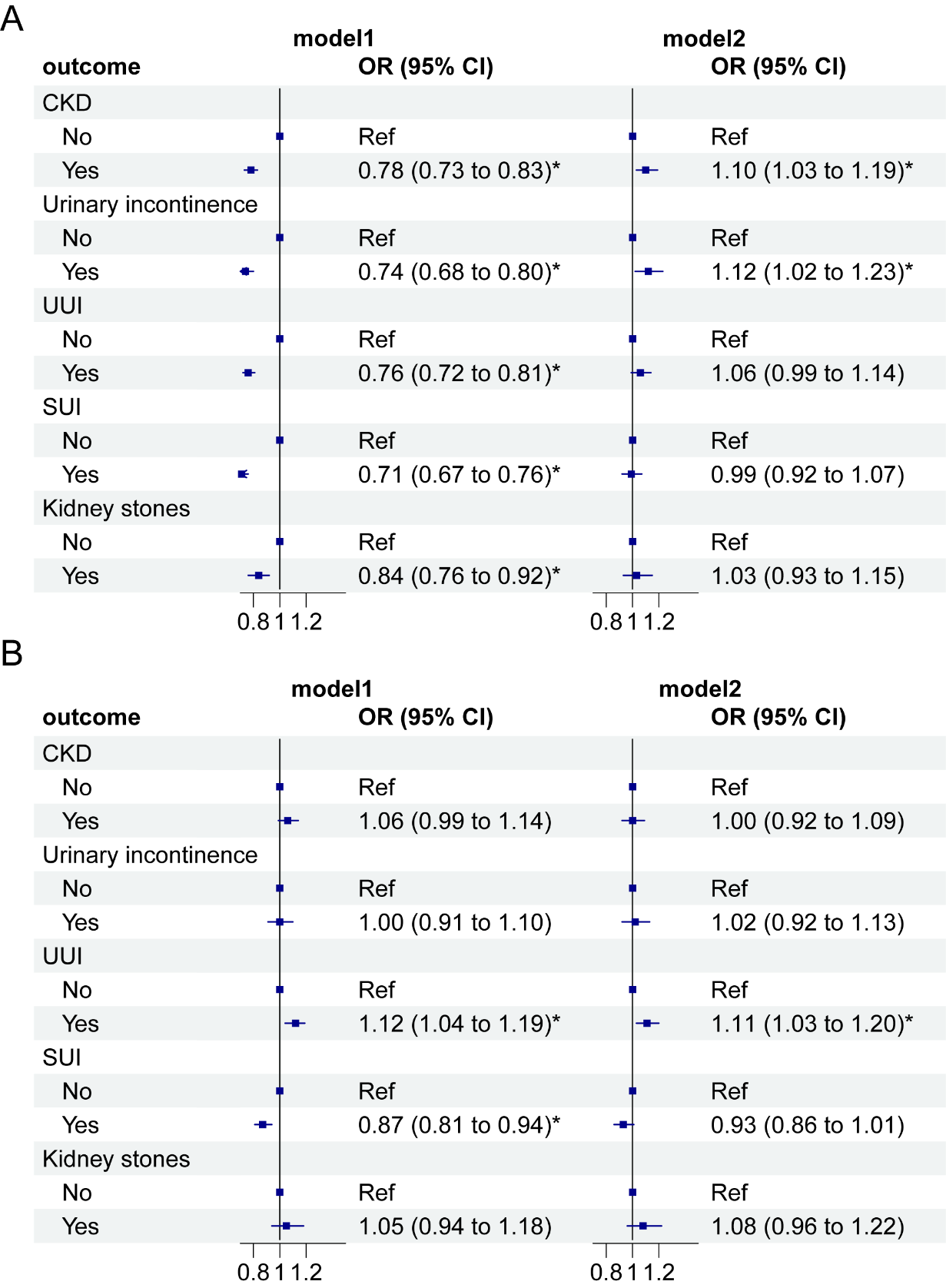
**Fig. S3. Associations between skipping breakfast (a) and skipping dinner (b) and urological diseases among U.S. adults (1999–2020) after excluding pregnant participants.**

Model 1 is unadjusted for covariates

Model 2 is adjusted for the following covariates: age, gender, race, family income level, education level, BMI, sedentary time, smoking status, calorie intake, sugar intake, liquid intake, calcium intake, protein intake, serum uric acid, and diabetes status

Abbreviations: CKD, chronic kidney disease; UUI, urge urinary incontinence; SUI, stress urinary incontinence; BMI, body mass index
